# Employee engagement during COVID-19 in Malaysia

**DOI:** 10.3389/fsoc.2022.976966

**Published:** 2022-11-07

**Authors:** Amira Mustaffa, Surianti Lajuma, Walton Wider

**Affiliations:** Faculty of Business and Communications, INTI International University, Nilai, Malaysia

**Keywords:** employee engagement, COVID-19, communication, training and development, transformational leadership, Malaysia

## Abstract

This objective of this study was to examine the effects of communication, training and development, and transformational leadership on employee engagement during COVID-19 in Malaysia. Four hundred individuals were recruited, and data were analyzed using partial least square structural equation modeling (PLS-SEM). Communication, training and development, and transformational leadership were found to positively affect employee engagement. In the midst of the COVID-19 outbreak, this study investigated the aforementioned factors as part of the reciprocal process between the employee and the employer and their effects on employee engagement, thereby making original theoretical contributions. This study also provides vital insights for businesses to consider when designing effective employee engagement plans for future well-being in the workplace.

## Introduction

The World Health Organization (WHO) declared the COVID-19 outbreak that struck China at the end of 2019 an international public health emergency that may threaten the country's health system, and, on 11 March 2020, the WHO declared the COVID-19 outbreak a pandemic (Firmansyah, 2021). The COVID-19 pandemic has caused a significant number of human casualties as well as worldwide economic recession, resulting in global devastation (Mirza et al., 2020; Fernández-Villaverde and Jones, 2022; Krueger et al., 2022; Lazebnik et al., 2022). Consequently, businesses must adapt their responses to the current pandemic environment. Human resource managers are constantly developing innovative, creative, and effective methods to engage employees in a healthy manner during this challenging period. Employee engagement is a workplace mentality that encourages all the employees in a company to give their best effort on a daily basis, committing to the organization's goals and values (Chanana and Sangeeta, 2020). In recent years, corporate leaders have grown concerned about the global economic downturn and the decline in employee engagement caused by the influx of millennials (Park, 2019). As a result of these circumstances, businesses are nowadays confronted with obstacles that have never been encountered before (Narula, 2020). It is therefore essential to study the topic of employee engagement if society is to overcome the current economic climate. The emotional connection between employees and an organization and its mission is referred to as employee engagement; a valuable asset to the organization due to its difficult-to-imitate strategic advantages (Raza et al., 2017; Hamadamin and Atan, 2019).

Engagement is a key concept in management, research, and practice, owing to its link with job performance, service climate, employment, and personal resources (Kosaka and Sato, 2020). Employee engagement was first defined as “personal involvement and self-reliance” by organizations concerned about and focused on profitability while at the same time seeking a competitive advantage in the market (Memon et al., 2020). Employee engagement is the process of developing an organizational ecosystem that allows employees to feel motivated in their work, while also demonstrating a high level of commitment to the organization by providing their best effort to perform any tasks or responsibilities assigned to them (Vijayalakshmi et al., 2021). Nowadays, organizations are continually developing innovative and effective means to engage employees during this challenging period (Chanana and Sangeeta, 2020).

Surviving in the corporate sector requires maximizing profits by utilizing existing capabilities (Osborne and Hammoud, 2017; Putra et al., 2021). Mansor et al. (2018) demonstrated that a statistically significant correlation exists between employee engagement and firm productivity, profitability, staff retention, safety, and customer satisfaction. The psychological pressure and uncertainty caused by a constantly changing workplace environment have negatively impacted workers (De-la-Calle-Durán and Rodríguez-Sánchez, 2021). Employees are important to an organization because they help to achieve profitability objectives, and company leaders must endeavor to engage employees in order to maintain this profitability (Osborne and Hammoud, 2017; Nguyen and Nguyen, 2020). If employees are engaged in their work, the performance and outcomes of an organization will improve (Lavigna and Basso, 2020). These are some of the most crucial factors for the success and survival of a business in challenging times (Van Der Voet and Vermeeren, 2017).

Employee engagement is a critical factor that drives employee performance, achievement, and consistent improvement throughout an entire year (Adhitama and Riyanto, 2020). Employee involvement in engagement activities in Malaysia is of major concern, with only 11% of employees showing involvement, 8% showing no involvement, and the remaining 81% showing less involvement (Jian et al., 2020). According to a recent study by Qualtrics, Malaysia has a higher rate of employee engagement compared with its global counterparts. The average Malaysian employee engagement score is 54%, higher than the global average of 53% (Mokhtar et al., 2021). According to a Gallup study on employee engagement in over 125 organizations, companies that invest in employee engagement can expect earnings to grow 2.6 times faster than companies with low employee engagement. These low-engagement companies experienced a 32% decline in operating income and an 11% decline in earnings per share growth (Juan and Yao, 2017).

Based on the aforementioned findings, employee engagement in Malaysia has significant room for improvement. Improved employee engagement in an organization will allow employees to understand their responsibilities within that organization and will encourage them to work with their colleagues to achieve the company's goals (Mansor et al., 2018). Thus, effective communication, the provision of training and development, as well as the attitude of company leaders, play significant roles in boosting employee engagement (Park, 2019).

This study aims to answer the research question: “What are the key factors for improved employee engagement during COVID-19 in Malaysia?” Answering this question is crucial because while numerous studies have been conducted on employee engagement in recent years (Uddin et al., 2019), studies on employee engagement during the COVID-19 pandemic in Malaysia are scarce. Using the Social Exchange Theory as a theoretical framework, three key factors are identified: communication, training and development, and transformational leadership.

## Theoretical underpinnings

This study employed the Social Exchange Theory (SET) (Mansor et al., 2018); the second most prevalently used theory to examine employee engagement. SET was introduced in 1964 by Blau, who examined the concept of “reciprocity,” which states that if organizations treat their employees with a kind, caring, and fair attitude, then employees will reciprocate the organization's kindness with similarly good behavior (Memon et al., 2020). This reciprocal relationship allows trust, loyalty, and commitment to develop over time, since, as Blau indicated, employee engagement and company commitment are interrelated (Sugandini et al., 2018). When an organization provides employees with sufficient resources and support, employee engagement increases (Ghasempour Ganji et al., 2021).

Previous research has demonstrated that SET can influence organizational behavior, particularly when employees have spent the majority of their lives at work. Rewards for their efforts such as training, development, compensation, and recognition, should be provided (Ibrahim et al., 2021). This theory was established in studies that examined the impacts of perceived justice, human resource development and identification, and ethical climate on employee engagement and satisfaction (O'Connor and Crowley-Henry, 2019). Ibrahim et al. (2021) argue that SET was developed to examine progress since this theory is the most effective way to explain social exchanges between employees and organizations in terms of behavior and results. The Social Exchange Theory is also utilized in numerous studies to forecast individual behavior (Uddin et al., 2019). The model provides a theoretical basis for comprehending why employees choose to be either more or less engaged at work (Wushe and Shenje, 2019). The Social Exchange Theory is also a method of rewarding an employer based on an employee's level of engagement (Ibrahim et al., 2021). SET explains how employees in an organization will exhibit engaged behaviors when leaders provide them with assistance and interest (Malik et al., 2017). An organization and its teams are largely influenced by its leaders, and the culture in an organization ultimately plays a significant role in determining the levels of commitment and engagement exhibited by its members (Mumford and Hunter, 2005). Additionally, SET proposes that employees who are highly engaged in both their job and their organization demonstrate greater affection toward their employer, as evidenced by higher levels of affective commitment. Engaged employees also feel an obligation to remain longer at a company, as evidenced by high levels of normative commitment and low levels of continuance commitment (Mokhtar et al., 2021). SET suggests that when positive internal communication occurs between an organization, the superiors, and the employees, the employees will view the relationship positively and will respond with engagement-related cognitions, behaviors, and emotions (Siddiqui and Sahar, 2019).

## Literature review

### Employee engagement

Engagement is defined as “the harnessing of organization members' selves to their work roles; in engagement, individuals use and express themselves [*sic*] physically, cognitively, and emotionally during role performances” (Mohamed Saad et al., 2018). The physical force expended in fulfilling an organizational role is referred to as a physical aspect; the cognitive component is linked to the beliefs of leaders and employees; and the emotional aspect refers to employees' positive or negative attitudes toward the organization and its leaders (Ibrahim et al., 2021).

Employee engagement is the psychological experience a person has that influences the emotional, physical, and cognitive aspects of their daily work processes and behaviors (Osborne and Hammoud, 2017). This statement is also supported by Ahmed (2019), who asserts that employee engagement is influenced by cognitive, emotional, and behavioral contexts. Others have defined employee engagement as individuals who are mentally present, focused, and who fully express themselves while performing organization-related tasks (Lai et al., 2020).

Due to its complexity and strict regulations, employee engagement will remain a challenge for many businesses in the future (Osborne and Hammoud, 2017). Employee engagement is important for both the employee and the organization because it encourages employee innovation and fosters the development of a close yet cordial relationship between management and employees (Bekirogullari, 2019). Employee engagement can also increase an organization's creativity, efficiency, and performance. It has a positive impact on employee performance and knowledge creation, which can provide financial returns to the organization, thereby reducing the cost of hiring and retaining employees (Ibrahim et al., 2021). Contract provisions can be utilized by employees to obstruct the achievement of company goals and objectives. Critical to the success of any business is the management's ability to implement employee engagement strategies (Osborne and Hammoud, 2017). Employee engagement is a positive state of mind; three traits characterize a positive and satisfying mindset: zeal, commitment, and concentration (Windia et al., 2020).

When an organization fosters a strong corporate culture in which employees feel valued and supported, business benefits such as cost- and time-savings result from positive employee engagement. Increased employee engagement stems from the management's trust in workers, flatter hierarchies, and leaders who serve as role models (Sievert and Scholz, 2017). Engagement has been associated with numerous positive organizational outcomes, including performance, profitability, and productivity (Bakker and Albrecht, 2018). Employee engagement is not synonymous with happiness, satisfaction, or well-being. Moreover, employee engagement is the degree to which employees experience mental and emotional connections to their workplace (Stange, 2020). Employee engagement has become one of the most urgent issues facing businesses in the twenty first century (Osborne and Hammoud, 2017). Firms should prioritize employee engagement in both good and bad times (Vickers, 2019). A number of additional important factors influence employee engagement, such as workplace well-being, organizational policies, procedures, structures, and systems (Ibrahim et al., 2021). Alzgoola et al. (2020) also included career development, training, leadership, teamwork, employee relations, compensation, and resources in that list. Psychology is likewise essential for improving engagement. According to Mokhtar et al. (2021), the majority of research conducted to date indicates that work engagement leads to organizational commitment, thereby creating employee engagement and increasing employee commitment. Notably, studies have demonstrated that when an organization's level of employee engagement is high, their performance improves, indicating a correlation between employee engagement and employee performance (Ibrahim et al., 2021).

This paper consists of three primary sections. In the first section, the relevant literature, concepts, and theoretical foundations are discussed. An outline of the most important findings in studies that are comparable with our study is provided. In the second section, the research methodology is described, followed by an analysis of the findings. Finally, conclusions and recommendations are provided based on these findings.

### Hypotheses development

#### Communication and employee engagement

Communication derives from the Latin word “communis,” which means “common.” Each individual's communication style is a unique combination of his or her innate abilities (Asuelimen and Omohimi, 2019). The foundation for any organization's success, whether small or multinational, is communication (Suparna, 2017). Asuelimen and Omohimi (2019) defined communication as the process of sending or transferring data, where the sender is the person who initiates the communication, and the receiver is the person who receives it. Interacting regularly without verbal or other forms of communication is almost impossible. Communication is the process of exchanging information between an organization's management and its employees; this is also known as internal communication, employee communication, staff communication, and internal public relations (Komodromos, 2020). Communication in an organization can affect the organization's performance, and if it is well-planned, it will increase employee satisfaction, facilitate information flow, and foster consensus (Martinez and Fernandez Hurtado, 2018). A well-planned and well-managed communication strategy can assist the management in establishing rapport with employees (Komodromos, 2020). Internal communication is essential for fostering and sustaining high employee engagement (Siddiqui and Sahar, 2019).

Moreover, communication skills are necessary for change-oriented leaders who want to express their influence in the most effective and meaningful manner possible when promoting organizational change (Othman et al., 2017). In recent years, communication within organizations and employee engagement have evolved in accordance with the modern information age, which is characterized by major technological breakthroughs and globalization (Komodromos, 2020). A study conducted by Eskelinen et al. (2017), based on the concepts of employee communication in an organization and employee engagement, concludes that organizations that utilize employee engagement techniques are able to increase employee motivation, promote communication, and foster agreement during problem solving. Consequently, the proposed hypothesis is stated as follows:

H1: *Communication positively affects employee engagement*.

#### Training and development and employee engagement

Providing employees with additional or new knowledge, skills, and training is associated with improved organizational outcomes and employee performance (Mansor et al., 2018). Organizations strive for differentiated programmes, services, capabilities, and products; however, well-trained professionals must imagine, design, implement, and maintain these characteristics (Rodriguez and Walters, 2017). Teaching new approaches, knowledge, and skills can help to retain employees because their jobs become more interesting, and they will be satisfied when genuine career development opportunities are available for managerial and professional staff (Mansor et al., 2018). Organizations are responsible for meeting the needs of its employees, such as providing proper training and a pleasant work environment, while employees are responsible for making a significant contribution to the organization based on the availability of training and the quality of the work environment (Osborne and Hammoud, 2017). This is further supported by Presbitero (2017); when employees are aware of their employers efforts to provide training and development, they are more likely to work toward achieving employment goals. The Social Exchange Theory also supports the notion that when employees believe they have been treated fairly by their employer, they will feel obligated to contribute to the organization's success (Uddin et al., 2019). Rodriguez and Walters (2017) argue that employee training and development is a crucial component of human resource planning because it can not only maximize individual returns but can also attract better employees.

Training is the systematic process of influencing an individual's knowledge, attitudes, and abilities for the purpose of personal development or future employment. Development is a systematic endeavor that influences an individual's knowledge, skills, and attitudes in order to promote personal growth or future employment opportunities (Presbitero, 2017; Khairul Zaman et al., 2021). Training and development is the process of improving an employee's performance in terms of skills, knowledge, attitude, and conduct. It is capable of enhancing individual and organizational superiority, as well as employee working skills (Chaudhry et al., 2017). Therefore, the proposed hypothesis is stated as follows:

H2: *Training and development positively affects employee engagement*.

#### Transformational leadership and employee engagement

In recent years, the global economic downturn and declining employee work levels have been linked to ongoing globalization and have become a topic of concern among organizational leaders. A variety of approaches are currently being considered to triumph over this challenging economic situation, and the concerns of organizational leaders are centered on leadership and employee engagement. According to Othman et al. (2017), leadership style is predicted to influence employee engagement. Employees and organizations are profoundly impacted by leadership in a variety of ways. Superiors motivate and engage subordinates through leadership, thereby enhancing their motivation and commitment to the organization (Malik et al., 2017). Prominent researchers in the field have provided numerous explanations for how leadership impacts motivation, thinking, behavior, and performance (Deconinck et al., 2018). When employees are well-supported by their supervisors, they will be more willing to invest in challenging tasks and to engage in their work (Mansor et al., 2018).

Leaders, particularly authentic leaders, play a significant role in influencing employee engagement; thus, the ability of leaders to communicate effectively is fundamental since clear communication can provide both a mission and a vision for employee development (Osborne and Hammoud, 2017). According to Yao et al.'s (2017) research, a leader who is communicative, honest, and fair will influence employees' willingness to work more diligently and to comprehend their work. Therefore, the proposed hypothesis is stated as follows:

H4: *Transformational leadership positively affects employee engagement*.

**Figure 1:**
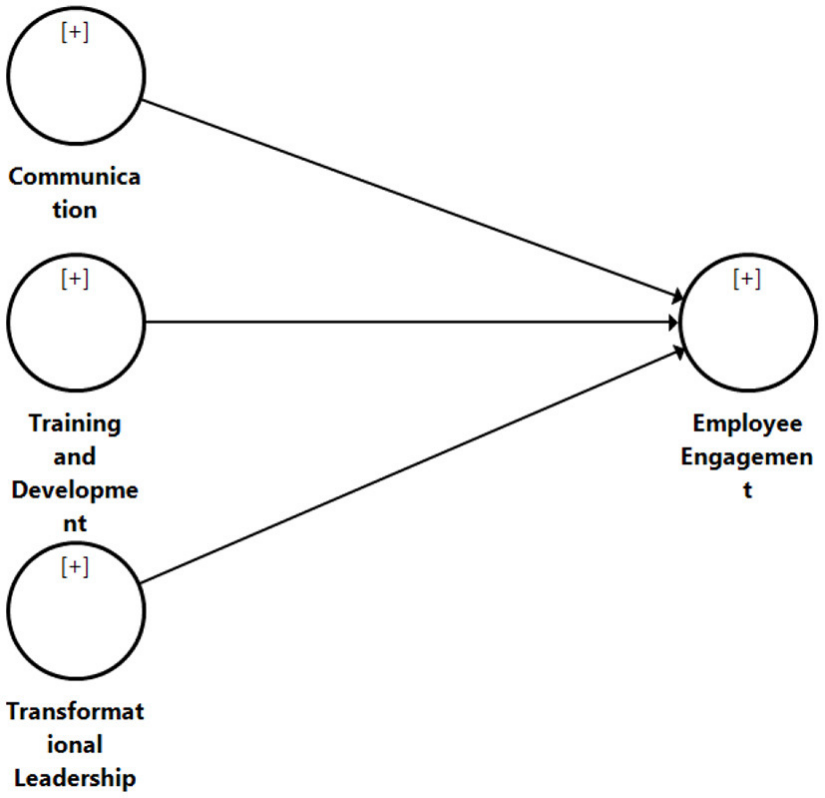
Conceptual framework.

Based on a review of both the relevant literature and the underlying theories, a conceptual framework was developed (refer to Figure 1).

## Method

### Research design

**Figure 2:**
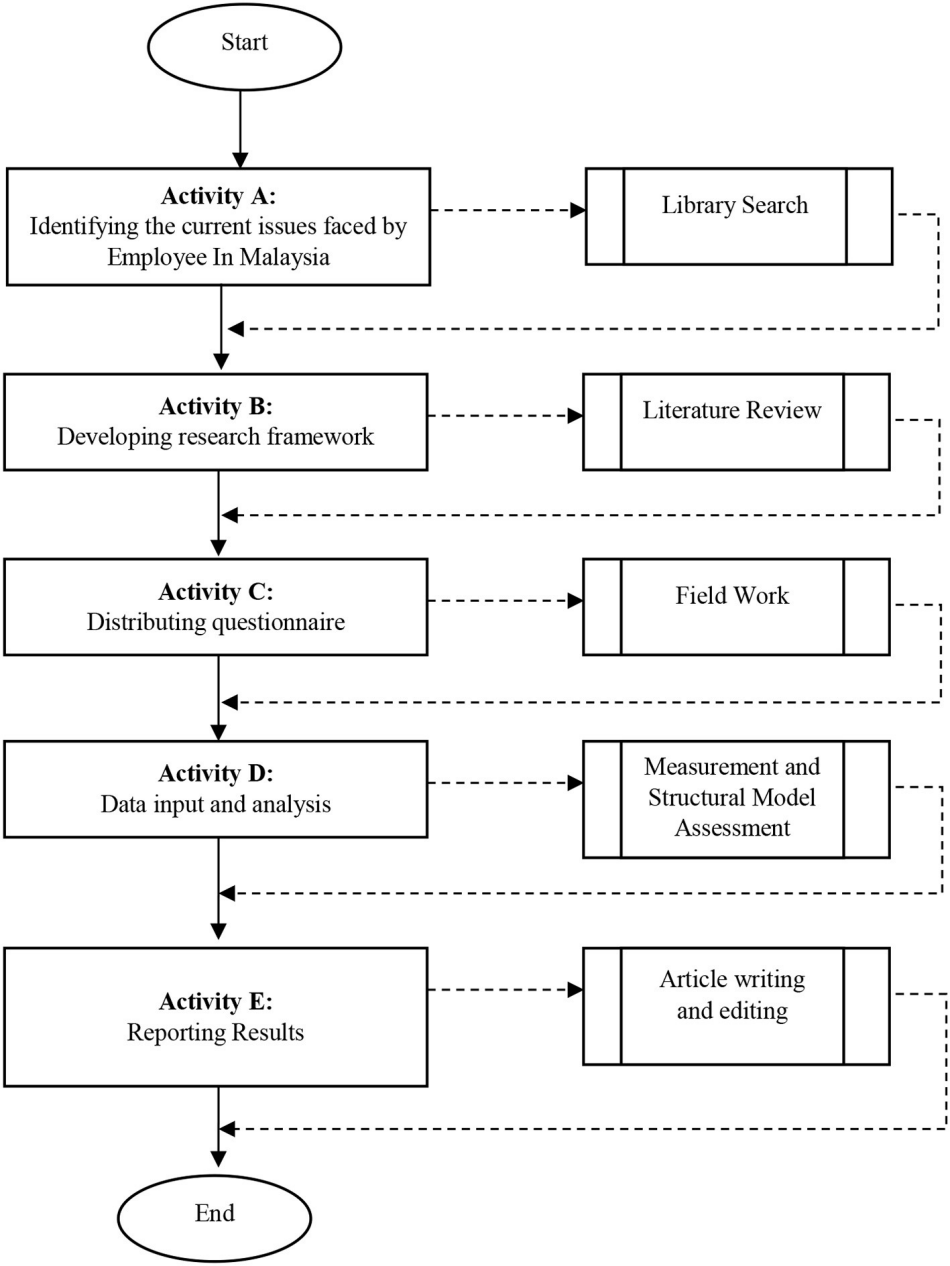
Flowchart of research methodology.

This cross-sectional study aims to explore employee engagement during the COVID-19 pandemic in Malaysia. The research methodology is shown in Figure 2. The primary method of data collection was through the distribution of questionnaires to appropriate target respondents. Google Forms was used to distribute a questionnaire comprising three sections: Section A (demographic), Section B (employee engagement), and Section C (communication, training and development, and transformational leadership).

### Population and sample

According to the Department of Statistics, Malaysia, the number of people in employment in Malaysia at the end of 2020 was ~15.22 million; these individuals serve as the sampling framework for this study. Since data collection was performed online (a link was generated in Google Forms) using social networking sites such as WhatsApp, Facebook, as well as by email, the questionnaire was sent to co-workers, family members, acquaintances, and a network of connections. The respondents were then asked, using the snowball technique, to propose other individuals that fell within the sampling framework who would be ideal candidates for the survey.

According to Krejcie and Morgan (1970), if the population of a country is higher than one million, the sample size should not be <384. Therefore, 400 questionnaires were sent to a diverse range of respondents in order to collect sufficient data.

### Data collection procedure

Upon study approval, a questionnaire in the form of an e-survey was sent using convenience sampling. Due to COVID-19, data were collected online and the respondents were informed of the purpose of the study prior to completing the survey. The e-survey also included information on the risks that the respondents may face (if any), the mutual benefits of participating in this study, and how their privacy and confidentiality would be protected throughout the duration of the study. The estimated time for a respondent to complete the questionnaire was between 8 and 10 min, depending on their level of comprehension. Respondents were given unrestricted time to complete the questionnaire. All respondents completed the questionnaire without any instances of missing data. All data collected were entered into an SPSS system used for data cleansing while SmartPLS 3.0 was used for data analysis.

Table 1 summarizes the demographic characteristics of the 400 respondents. The five primary components of the demographic section were gender, age, highest qualification, tenure in the current organization, and position level. Sixty-two per cent of respondents were female, while the remaining 38% were male; 58% of the respondents held a bachelor's degree as their highest level of education and the majority of respondents (43.5%) were between the ages of 31 and 40. Regarding their positions, 205 of the respondents (51.3%) were in middle management and the majority of respondents (32.3%) were employed by their organization for between 1 and 5 years.

**Table 1:** Demographic profile of respondents.

**Variable**	**Classification**	**Frequency**	**Percentage (%)**
Gender	Male	152	38.0
	Female	248	62.0
Age	Under 20 years of age	7	1.8
	20–30 years of age	153	38.3
	31–40 years of age	174	43.5
	41–50 years of age	55	13.8
	51–60 years of age	10	2.5
	More than 60 years	1	0.3
Highest qualification	SPM/STPM	29	7.2
	Certificate/Diploma	99	24.8
	Bachelor's degree	232	58.0
	Master's degree	32	8.0
	Ph.D.	8	2.0
Length of employment in current organization	Less than 1 year	47	11.8
	1–5 years	129	32.3
	6–10 years	118	29.5
	11–15 years	62	15.5
	16–20 years	31	7.8
	Over 20 years	13	3.3
Current position	Senior management	62	15.5
	Middle management	205	51.3
	Entry level	133	33.3

### Measures

#### Employee engagement

Employee engagement was measured using the 9-item Utrecht Work Engagement Scale (UWES) (Schaufeli et al., 2002), with a 5-point Likert scale ranging from 1 (strongly disagree) to 5 (strongly agree). Respondents were asked to evaluate their level of engagement in their organization based on three factors: vigor, which refers to high levels of energy; resilience; and a willingness to confront challenges. One of the statements regarding vigor is “At my job, I feel strong and vigorous.” “Dedication” refers to a worker with a sense of pride in the work done, and this section includes the statement “I am proud of the work that I do.” “Absorption” refers to an employee who is so fully and happily immersed in work and/or pleasure that he or she is unaware of the passage of time while performing a task. This section contains the statement “When I'm working, I forget everything else around me” (Schaufeli and Bakker, 2003). Previous research performed by Ibrahim et al. (2021) revealed high consistency values for vigor (0.84), dedication (0.86), and absorption (0.82).

#### Communication

This construct consisted of six items that were obtained from Botero and Van Dyne (2009) and placed on a 5-point Likert scale. The scale ranges from 1 (strongly disagree) to 5 (strongly agree). The following is an example of a statement in this section: “I communicate my opinions about work-related issues to others in my work unit, even if their opinions differ from mine and they disagree with me.” The value of the Cronbach alpha was 0.96.

#### Training and development

This construct comprised six items adapted from Siddiqui and Sahar (2019) and utilized a 5-Point Likert Scale ranging from 1 (strongly disagree) to 5 (strongly agree). An example of a statement in this section is as follows: “Staff training allows employees to proactively identify future challenges.” The value of the Cronbach alpha was 0.95.

#### Transformational leadership

This construct comprised 10 items adapted from Rafferty and Griffin (2004) 5-Point Likert Scale, ranging from 1 (strongly disagree) to 5 (strongly agree). An example of a statement in this section is as follows: “Encourages individuals to view changing environments as situations ripe with opportunity.” The value of the Cronbach alpha was 0.98.

### Data analysis

This primary objective of this study was to examine the impact of communication, training and development, and transformational leadership on employee engagement. Therefore, we utilized PLS-SEM owing to its prediction-oriented approach (Hair et al., 2022), which aligns well with the purpose of our investigation. PLS-SEM is a comprehensive analytical method that uses the SmartPLS 3.0 software to concurrently evaluate both the measurement model and the structural model (Ringle et al., 2014; Legate et al., 2021). The associations between latent variables and the related measurement items or indicators were assessed in order to evaluate the measurement model. Indicator reliability and validity were also utilized to evaluate the appropriateness of the measures included in this study. The reliabilities of various indicators, the internal consistency reliabilities of each construct, as well as the convergent and discriminant validities were included in the procedure (Legate et al., 2021). To evaluate the structural model, path coefficient analysis was conducted to describe the link between each latent variable. The model evaluated the endogenous variables (*R*^2^), the path coefficient estimation, and a 95% confidence interval (CI 0.95; Hair et al., 2022).

### Assessment of the measurement model

Firstly, it was necessary to assess the construct reliability and validity of the measurement model. The measurement model consisted of the following four reflective constructs: communication, training and development, transformational leadership, and employee engagement. The factor loadings, composite reliability (CR), and Rho_A values should be >0.70 (Ali et al., 2018). However, items with factor loadings between 0.4 and 0.7 are retained if the CR and AVE of that particular variable are greater than the cutoff value (Hair et al., 2022). Table 2 shows that all items were loaded into their own construct and exceeded the cutoff value of 0.7 for both CR and Rho_A. Following this, the study's convergent validity was assessed using the item factor loading and the average variance extracted (AVE). Henseler et al. (2009) defined convergent validity as a collection of indicators that represent the same or similar underlying constructs. According to Hair et al. (2022), the AVE threshold value should exceed 0.50 as a general rule of thumb. Table 2 shows that all constructs exhibited an adequate AVE value and that each construct can explain more than half of its variance. Thus, the thresholds for construct reliability and convergent validity were reached.

**Table 2:** Results for the assessment of the measurement model.

**Latent variable**	**Item**	**Loading**	**AVE**	**CR**	**Rho_A**
Employee engagement	EE1	0.876	0.733	0.961	0.958
	EE2	0.850			
	EE3	0.831			
	EE4	0.882			
	EE5	0.935			
	EE6	0.915			
	EE7	0.915			
	EE8	0.782			
	EE9	0.695			
Communication	COM1	0.929	0.837	0.968	0.964
	COM2	0.930			
	COM3	0.916			
	COM4	0.924			
	COM5	0.866			
	COM6	0.922			
Training and development	TD1	0.869	0.808	0.962	0.952
	TD2	0.895			
	TD3	0.868			
	TD4	0.933			
	TD5	0.906			
	TD6	0.919			
Transformational leadership	TL1	0.854	0.836	0.981	0.978
	TL2	0.926			
	TL3	0.899			
	TL4	0.920			
	TL5	0.859			
	TL6	0.929			
	TL7	0.932			
	TL8	0.951			
	TL9	0.953			
	TL10	0.915			

Secondly, it was necessary to establish the discriminant validity of the constructs in this study. For this we employed the heterotrait-monotrait (HTMT) ratio (Ghasemy et al., 2020). Discriminant validity is established when the HTMT ratio is <0.90 (Hair et al., 2019). Table 3 shows that the HTMT values for all constructs were less than 0.90, confirming the discriminant validity of this study's model.

**Table 3:** Discriminant validity using the HTMT ratio.

	**Communication**	**Employee engagement**	**Training and development**	**Transformational leadership**
Communication	0.915			
Employee engagement	0.726	0.856		
Training and development	0.755	0.729	0.899	
Transformational leadership	0.693	0.776	0.8	0.914

### Structural model assessment

Before conducting the assessment, the collinearity between the research variables in the structural model was evaluated to ensure that there was no issue with lateral collinearity (Hair et al., 2022). Table 4 shows that all inner VIF values were <5 (Hair et al., 2022), indicating that there were no collinearity issues. In addition, the bootstrap method was used on 5,000 resamples to evaluate the path coefficients of the structural model. The t-values and the 95% bias-corrected confidence intervals were also evaluated in order to determine the sign and significance of the path coefficients (Hair et al., 2019). The results reveal an *R*^2^ value for employee engagement of 0.675, which is regarded as high in behavioral studies (Hair et al., 2019). The findings indicate that communication (β = 0.312, *t* = 5.189, and *p* < 0.01) and transformational leadership (β = 0.459, *t* = 7.725, and *p* < 0.01) positively influenced employee engagement, thereby supporting hypotheses H1 and H3. Although training and development exhibited a significant *t*-value (β = 0.127, *t* = 2.078, and *p* < 0.05), the 95% bias-corrected confidence interval fell between 0 and 1, indicating that the H2 hypothesis was not supported. Based on the path coefficient, transformational leadership had the greatest impact on employee engagement, followed by communication.

**Table 4:** Results of the hypothesis testing.

**Hypothesis**	**Relationship**	**β**	***t*** **-value**	**95% CI**	***R*** **^2^**	**Results**	**VIF**
H1	Communication → Employee engagement	0.312	5.189	[0.194, 0.430]	0.675	Supported	2.449
H2	Training and development → Employee engagement	0.127	2.078	[−0.003, 0.241]		Not supported	3.531
H3	Transformational leadership → Employee engagement	0.459	7.721	[0.342, 0.573]		Supported	2.925

## Discussion

This study seeks to determine the impact of communication, training and development, and transformational leadership on employee engagement. The strongest indicator of employee engagement was determined to be transformational leadership, followed by communication. Training and development, however, were found not to be significant predictors of employee engagement.

The result from first hypothesis of this study, H1, is that communication positively affects employee engagement, which is consistent with the findings of Jha et al. (2019), who discovered that communication has a positive and significant relationship with employee engagement. Communication can increase both an employee's engagement and their interaction with a job, in which they contribute ideas and thoughts to help the organization achieve its goals and objectives. This suggests that improved communication can motivate employees to concentrate on enhancing their work processes and organizing their thoughts and opinions while pursuing their performance objectives in accordance with company standards (Ibrahim et al., 2021). Keating (2015) discovered that effective communication fosters staff engagement and commitment, which are associated with increased profitability, productivity, and customer satisfaction. According to previous research, employees will feel more engaged when they can communicate face-to-face with management (Komodromos, 2020). Ideally, organizations should provide appropriate channels for communicating with employees so that information can be communicated swiftly and immediately, particularly in the event of a pandemic. Personnel want their message to reach as many people as possible; therefore, multimedia distribution is the optimal method.

In accordance with Balwant et al. (2019) the result from the third hypothesis of this study is that transformational leadership positively influences employee engagement. A clear leader's vision will assist employees in achieving company objectives while appreciating the value of their dedication to achieving objectives. According to (Chanana and Sangeeta, 2020), leadership must be more apparent during difficult times than at any other time. If companies want their employees to be engaged, their leaders must assume responsibility and should inspire employees to fulfill their future commitments. Moreover, transformational leaders can motivate their employees to alter their lifestyles in order to accomplish more expansive missions and visions. Ibrahim et al. (2021) argue that a leader's positive attitude and behavior will instill inspirational attitudes in employees. SET emphasizes the social structures that are developed through repeated exchanges, as well as the manner in which these structures either limit or enable leaders to exert power and influence (Cook et al., 2013).

The result from the second hypothesis of this study, H2, is that training and development has no positive effect on employee engagement. This contradicts a previous study which showed that training and development positively affects employee engagement (Ibrahim et al., 2021). Our result also contradicts Afroz (2018), whose findings revealed that respondents had regularly engaged in training programs provided by their employer. Moreover, the majority of competent employees believed that their involvement in training programmes made them more devoted, fulfilled, and inspired. Although our findings did not reveal a positive relationship between training and development and employee engagement, we believe that training and development can assist employees in achieving organizational goals by improving their knowledge, skills, and attitudes. The second most important factor to engage employees, according to Gargantini et al. (2022), is a risk-free work environment in which they are allowed to experiment and learn from their mistakes. Therefore, training, development, and learning can be viewed as an internal motivator that helps employees to grow and to improve their individual development plans using rewards such as autonomy, relatedness, and competence. A good organization's training and development programme will provide effective, ongoing feedback to its employees, demonstrating that the company supports them.

In light of the results discussed in this study, organizations should consider the most effective means of sustaining employee engagement. Our findings show that factors such as communication and transformational leadership are key to successful employee engagement. An organization's employees are a valuable asset; they spend a significant portion of their lives working. Organizations require a minor adjustment to efforts that have already been made; for example, to training, development, rewards, recognition, and a pleasant work environment. Consequently, increased employee participation in an organization can not only increase the effectiveness of the organization, but can also improve job satisfaction (Jha et al., 2019; Ibrahim et al., 2021).

Since the theories discussed in this study have a positive correlation with employee engagement, organizations should consider various methods to improve both communication and transformational leadership in order to increase employee engagement during a pandemic. The American Management Association argues that companies can increase employee engagement, even during challenging times, if they care and make the right decisions at the right time (Chanana and Sangeeta, 2020). According to Komodromos (2020), employees must be involved in every step of strategic communication in order to reduce ambiguity, fear, and lack of confidence. This is because consistent communication can increase employee trust and can influence their behavior; making employees' expectations more realistic, thereby enhancing their sense of safety. Research has shown that underutilized communication and transparency strategies, as well as the use of evasion and cohesiveness approaches, affect employee trust and engagement (Mazzei and Ravazzani, 2014; Mishra et al., 2014). Therefore, internal communication and transparency are required to facilitate strategy transformation in medium-sized businesses. In the event of a strategic shift within an organization, the top management should improve two-way communication and should ensure that employees have access to all resources necessary to carry out their responsibilities. During a strategic shift, employees may protest against management activities if they are repressed as a result of management decisions (Komodromos, 2020). During the COVID-19 pandemic, a large number of employees were compelled to work from home, inevitably encountering communication issues due to internet outages and delays in information delivery. Of course, there are also positive outcomes. To ensure that their employees are satisfied and committed, organizations must implement innovative employee engagement strategies. During this difficult pandemic period, the measurement of employee engagement is crucial.

Furthermore, transformational leadership is an critical factor that influences employee engagement. According to Park (2019), coaching, advocating, providing performance evaluations, serving as a sounding board for career aspirations, and allotting sufficient time for development opportunities are examples of supervisory support. A transformational leader serves not only as a coach or mentor, but also helps each employee to develop and grow. Transformational leaders must develop their subordinates' expertise by appropriately guiding and supporting their short-, medium-, and long-term career development. Not only that, but leaders are also encouraged to show concern and respect for subordinates; this is directly related to the degree to which employees are satisfied with their leaders as well as their engagement at work.

### Limitations and future research

Using Social Exchange Theory, the present study supports earlier studies and clarifies the roles of communication and transformational leadership in employee engagement in Malaysia. There has been a recent need for theoretical research pertaining to the reciprocal relationship between an employee and their organization (Zainal et al., 2022). As such, from a theoretical standpoint, our study has bridged the information gap regarding the development of an optimal framework for employee engagement. The findings of our study will aid future researchers who may choose to pursue the subject further. Based on our results, communication and transformational leadership are positively linked to employee engagement during COVID-19 in Malaysia. Thus, and even after COVID-19, future studies may focus exclusively on one of the independent variables mentioned in this study in order to better and more thoroughly understand the impact on employee engagement.

This research is subject to a number of limitations, all of which need to be taken into consideration. Firstly, this study employs cross-sectional data. Since it is challenging to establish a causal relationship using a cross-sectional study, future researchers should analyse causal linkages by employing longitudinal studies. A qualitative method may provide respondents with additional opportunities to share diverse viewpoints that reflect more realistic points of view. Secondly, another limitation of this research that must be addressed is that data collection was completed online at the time, since Malaysia was under the Movement Control Order.

Future research could be conducted at specific locations in Malaysia's largest cities, including Kuala Lumpur, Johor Bahru, Penang, and the Klang Valley. Additionally, researchers could analyse various industries such as the education, services, food and beverages, and banking industries, as well as many others. Different cities and industries may have distinct cultures; thus, the findings may be more precise and applicable to specific regions.

## Conclusion

Employers must give workers the freedom to make their work interesting and must create an atmosphere where employees can live healthy work lives. Employees are a valuable asset to any organization, and if they are not given sufficient space and time to achieve the ideal balance between work and enjoyment, they will develop a feeling of disengagement. Organizations and employees depend on each other to achieve their respective objectives. Therefore, employee engagement should not be a one-time event, but should be ingrained in the company's culture. Effective communication and transformational leadership should be included in the process of employee engagement. Therefore, modern businesses must actively plan ahead to meet employee expectations and, as a result, influence employee engagement, which directly impacts organizational success.

## Contribution/Originality

The findings of this research will aid organizations in comprehending certain key factors that may improve employee engagement during a crisis situation. The findings of this research may be utilized when management decides to boost employee involvement both during COVID-19 and post-pandemic; by exploiting components of communication, training and development, or transformational leadership.

## Data availability statement

The raw data supporting the conclusions of this article will be made available by the authors, without undue reservation.

## Ethics statement

Ethical approval was not provided for this study on human participants because the study was conducted according to the guidelines of the Declaration of Helsinki and following academic ethics. The patients/participants provided their written informed consent to participate in this study.

## Author contributions

All authors listed have made a substantial, direct, and intellectual contribution to the work and approved it for publication.

## Conflict of interest

The authors declare that the research was conducted in the absence of any commercial or financial relationships that could be construed as a potential conflict of interest.

## Publisher's note

All claims expressed in this article are solely those of the authors and do not necessarily represent those of their affiliated organizations, or those of the publisher, the editors and the reviewers. Any product that may be evaluated in this article, or claim that may be made by its manufacturer, is not guaranteed or endorsed by the publisher.
