# Supplementary material for: Employee engagement during COVID-19 in Malaysia
Source: Front Sociol. 2022 Nov 7;7:976966. doi: 10.3389/fsoc.2022.976966 (PMC9676451; doi:10.3389/fsoc.2022.976966)
Supplement: Supplementary file 1 [file Data_Sheet_1.pdf]

Appendix

1. Plot

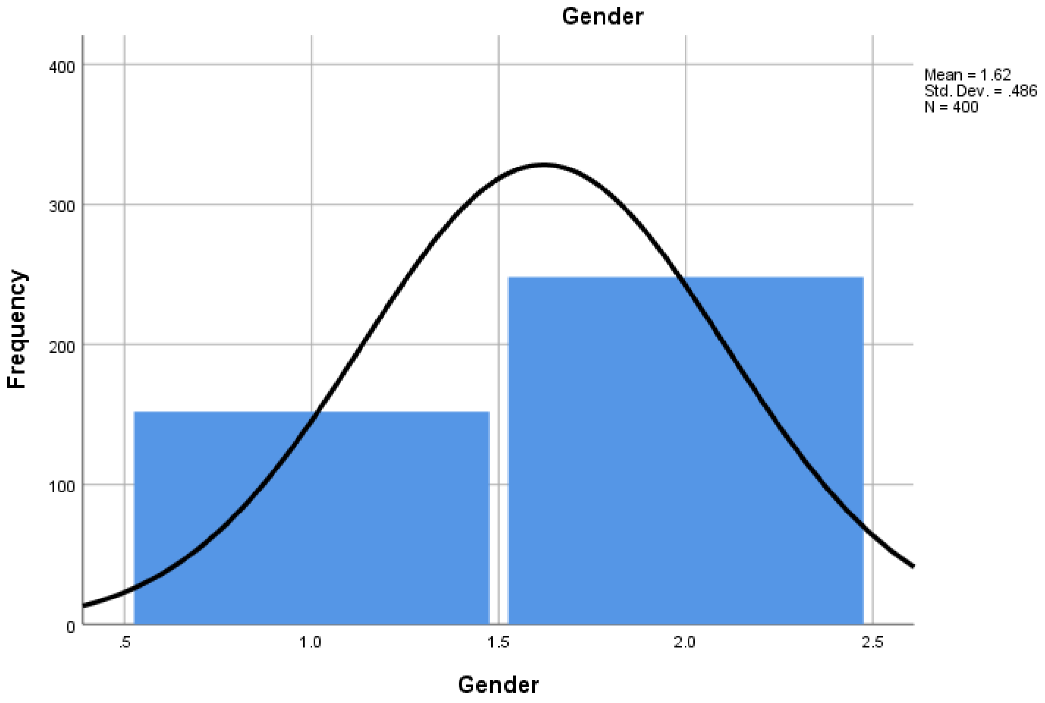

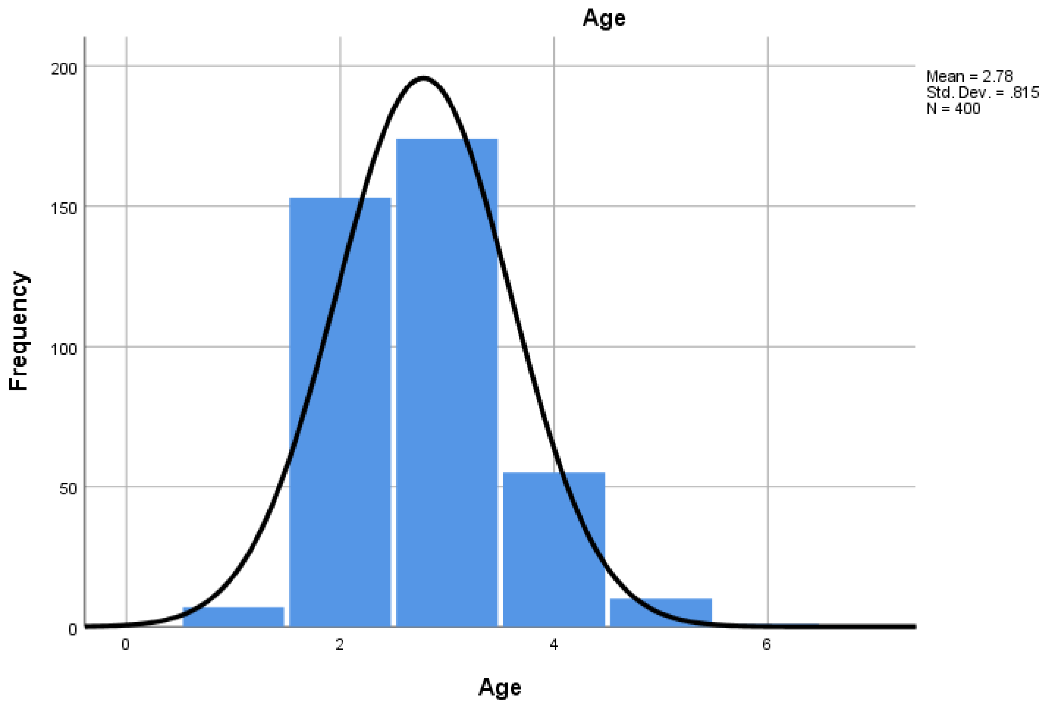
Age
Mean = 2.78
Std. Dev. = .815
N = 400
Frequency
0
50
100
150
200
0
2
4
6
Age

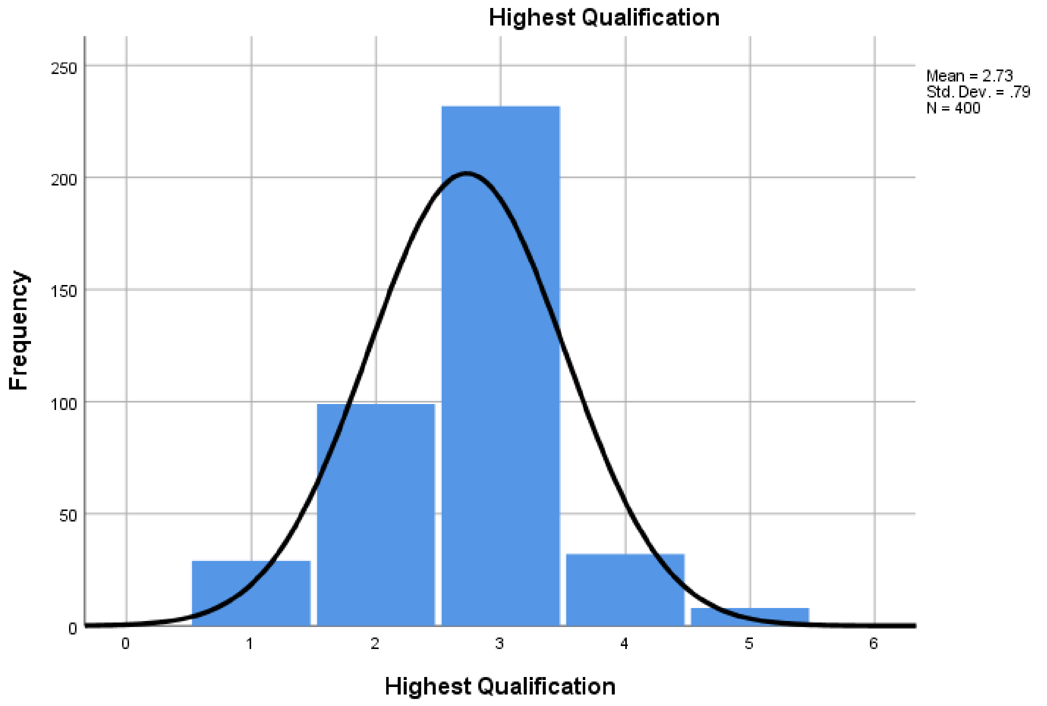
Highest Qualification
Mean = 2.73
Std. Dev. = .79
N = 400
Frequency
0
50
100
150
200
250
0
1
2
3
4
5
6
Highest Qualification

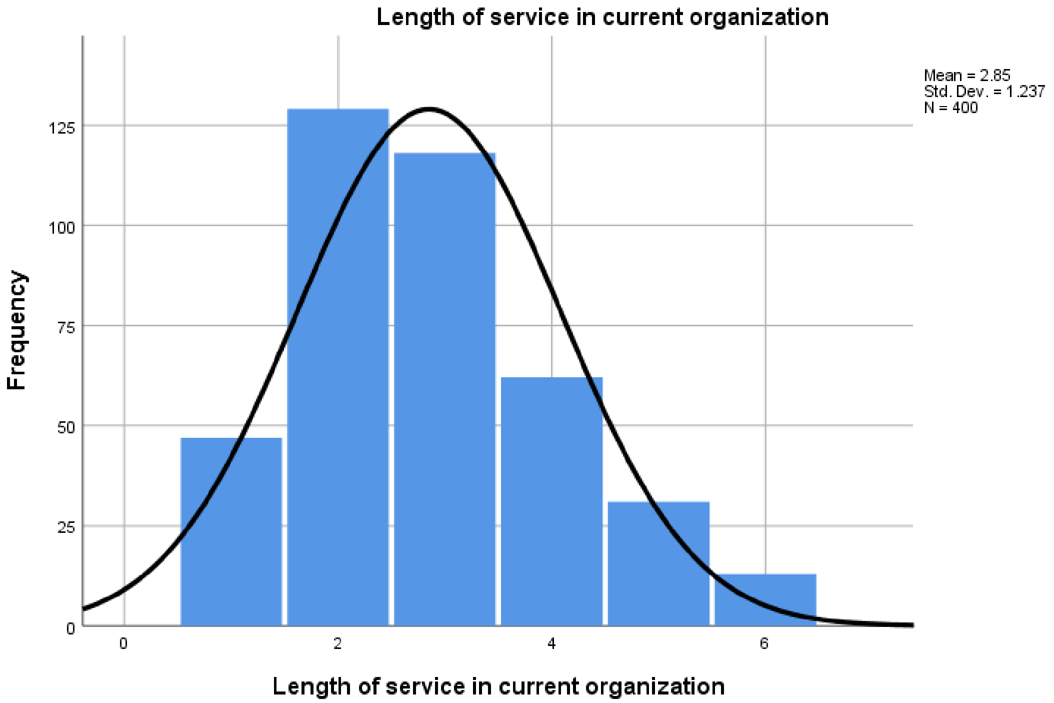
Length of service in current organization
Mean = 2.85
Std. Dev. = 1.237
N = 400
Frequency
125
100
75
50
25
0
0
2
4
6
Length of service in current organization

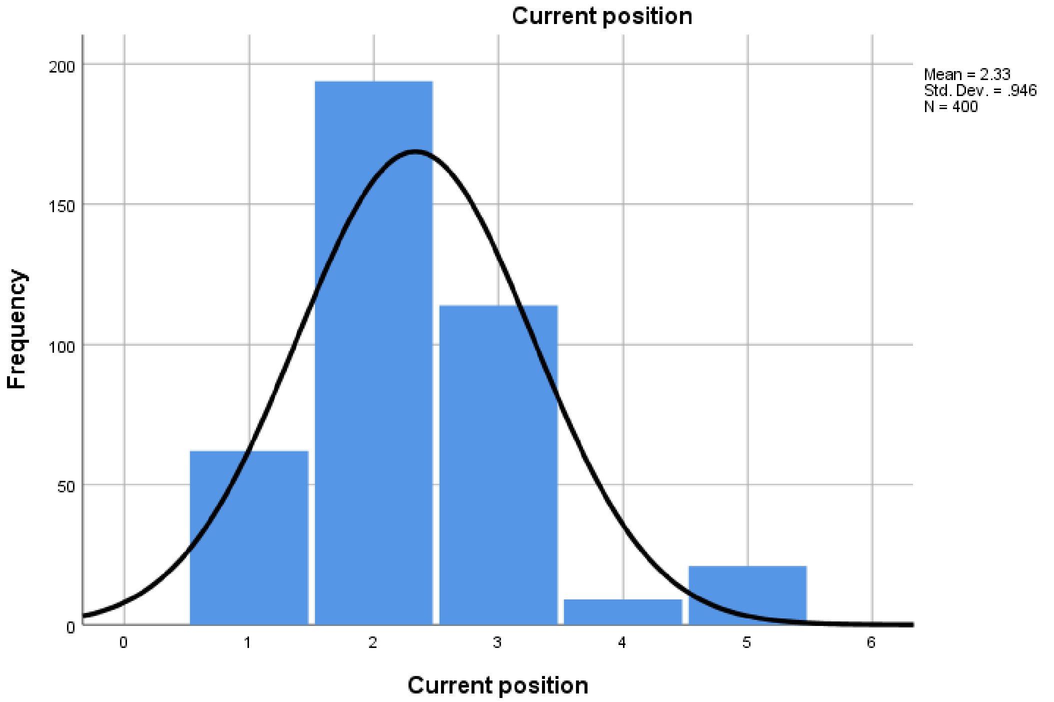
Current position
Mean = 2.33
Std. Dev. = .946
N = 400
Frequency
200
150
100
50
0
0
1
2
3
4
5
6
Current position

2. Measures and scales

Employee Engagement During COVID-19 in Malaysia

Dear Sir/Madam,

I would like to thank you for taking part in this survey and sparing your time to answer this survey. I am a student in INTI International University, Nilai and would like to conduct research on "Employee Engagement During COVID-19 in Malaysia" for my Master in Business Administration (MBA) project.

You are invited to take part in a research study. The aim of this study is to identify the factors that influence Employee Engagement During COVID -19 in Malaysia. Participation in this questionnaire is voluntary and respondents are guaranteed confidentiality and anonymity.

The purpose of this research is purely academic, and focus will only be driven towards what the results indicate rather than self-interest. The data analysis will be conducted in an ethical manner; considering the interests of all parties involved. The only thing that will cost you is your time. Your name will not be recorded to preserve your anonymity. Your privacy would be retained, and no information obtained from this study shall be sold to third parties for commercial purpose or disclosed in any manner that would identify you.

Without your cooperation and support, this research cannot be accomplished. We highly appreciated and thank you in advance for your time, involvement, and cooperation to participate in this survey. Your valuable input would be very helpful for the course of this study.

Thank you very much.

If you would like further information or would like to discuss any details, please get in touch with me, in writing, by phone or by email:

Researcher: Amira Binti Mustaffa
Phone: +6012-7704654
Email: i19017484@student.newinti.edu.my

Specific instruction is given at the beginning of each section of the questionnaire. Kindly complete the questionnaire by answering all questions in each section. This survey is done based on a voluntary basis. The respondents have all the rights to reject and choose not to answer the questions.

Section A: Demographic Information

Gender *
□Male
□Female

Age *
□Below 20 years
□20 - 30 years
□31 - 40 years
□41 - 50 years
□51 - 60 years
□Above 61 yeras

Highest Qualification *
□SPM / STPM
□Certificate / Diploma
□Bachelors Degree
□Masters
□PhD

Length of service in current organisation *
□Less than 1 year
□1 - 5 years
□6 - 10 years
□11 - 15 years
□16 - 20 years
□More than 20 years

Current position *
□Senior Management
□Middle Management
□Entry Level

Section B: Employee Engagement

The statement in this section is related to employee retention.

Please choose the number that best reflects your opinion about the statement using 5 Likert scale which that [(1) = strongly disagree, (2) = disagree, (3) = neutral, (4) = agree and (5) = strongly agree]

1	At my job, I feel strong and vigorous	1	2	3	4	5
2	At my work, I feel bursting with energy	1	2	3	4	5
3	I can continue working for very long periods of time	1	2	3	4	5
4	My job inspires me	1	2	3	4	5
5	I am enthusiastic about my job	1	2	3	4	5
6	I am proud of the work that I do	1	2	3	4	5
7	I find that the work that I do is full of meaning and purpose	1	2	3	4	5
8	Time flies when I am working	1	2	3	4	5
9	It is difficult to detach myself from work	1	2	3	4	5

Section C: Factors influencing employee engagement

Below listed are the series statements under the factors that influence employee retention. Please choose only one answer that best reflects your opinion and indicate each statement by making in the box provided below. Rate each statement using 5-point Likert Scale below: [1=Strongly Disagree, 2= Disagree, 3= Neutral, 4 = Agree, 5 = Strongly Agree]

Communication

1	I develop and make recommendations to my supervisor concerning issues that effect my work	1	2	3	4	5
2	I speak up and encourage others in my work unit to get involved in issues that effect our work	1	2	3	4	5
3	I communicate my opinions about work issues to other in my	1	2	3	4	5

	work unit, even if their opinions are different and they disagree with me					
4	I keep well informed about issues at work where my opinion can be useful	1	2	3	4	5
5	I get involved in issues that affect the quality of life in my work unit	1	2	3	4	5
6	I speak up to my supervisor with ideas for new projects or changes in procedure of work	1	2	3	4	5

Training and development

1	This organisation is much concern about my career development and growth Opportunities for the future by performance management	1	2	3	4	5
2	Training programs provided by the organisation are adequate for my development	1	2	3	4	5
3	Staff training allows employees to proactively identify future challenges	1	2	3	4	5
4	In my organisation employees adapt quickly to difficult situation due to training	1	2	3	4	5
5	With training, I am completely focused on my work	1	2	3	4	5
6	I am now more involved in my work and days goes by very quickly due to training	1	2	3	4	5

Transformational leadership

1	My leader has a clear understanding of where we are going	1	2	3	4	5
2	My leader says things that make employees proud to be a part of this organisation	1	2	3	4	5
3	My leader encourages people to see changing environments as situations full of opportunities	1	2	3	4	5
4	My leader challenges me to think about old problems in new ways	1	2	3	4	5
5	My leader has challenged me to rethink some of my basic assumptions about my work	1	2	3	4	5
6	My leader considers my personal feelings before acting	1	2	3	4	5
7	My leader behaves in a manner which is thoughtful of my personal needs	1	2	3	4	5
8	My leader sees that the interests of employees are given due consideration	1	2	3	4	5
9	My leader acknowledges improvement in my quality of work	1	2	3	4	5
10	My leader personally compliments me when I do outstanding work	1	2	3	4	5
